# Factors Related to Febrile Seizures Among Children at Abdul Moeloek Regional Hospital in Lampung Province

**DOI:** 10.21315/mjms-05-2025-369

**Published:** 2025-10-31

**Authors:** Roro Rukmi Windi Perdani, Ardina Marista, Dyah Wulan Sumekar Rengganis Wardani, Suharmanto Suharmanto, Tri Umiana Soleha

**Affiliations:** 1 Department of Paediatrics, Faculty of Medicine, Universitas Lampung, Bandar Lampung, Indonesia; 2 Medical Programme, Faculty of Medicine, Universitas Lampung, Bandar Lampung, Indonesia; 3 Department of Public Health, Faculty of Medicine, Universitas Lampung, Bandar Lampung, Indonesia; 4 Department of Microbiology and Parasitology, Faculty of Medicine, Universitas Lampung, Bandar Lampung, Indonesia

**Keywords:** children, febrile seizures, risk factors

## Abstract

**Background:**

Febrile seizures are a significant neurological condition that affects children between the ages of 6 and 60 months and can lead to long-term neurological outcomes, such as neurological, cognitive, and memory impairments. Despite their high global incidence, contributing factors and regional variations are not fully understood, particularly in Indonesia.

**Methods:**

A case−control study was conducted at Abdul Moeloek Regional Hospital in Lampung Province, Indonesia, to identify factors associated with febrile seizures among children under five years of age. A total of 72 children with fevers were selected through consecutive sampling in December 2020. Data on body temperature during seizures, fever duration, gender, age, family history of seizures, and birth weight were collected through structured interviews and medical records.

**Results:**

Statistical analyses included Chi-square tests and multivariate logistic regression. The results indicated significant associations between febrile seizures and body temperatures exceeding 39°C (*P* = 0.047; OR = 4.00), fever duration ≤24 hours (*P* = 0.030; OR = 3.35), male gender (*P* = 0.010; OR = 4.96), positive family history of seizures (*P* = 0.001; OR = 7.75), and low birth weight < 2,500 grams (*P* = 0.017; OR = 3.64). Multivariate analysis confirmed family history of seizures (β = 2.285, *P* = 0.001), male gender (β = 1.954, *P* = 0.005), and elevated body temperature (β = 1.525, *P* = 0.045) as independent risk factors.

**Conclusion:**

Febrile seizures are significantly associated with a positive family history of seizures, male sex, and elevated body temperature. Awareness of these risk factors may improve early recognition and management, ultimately improving paediatric neurological health

## Introduction

Febrile seizures are the most common seizure disorder in children and predominantly affect those between the ages of 6 and 60 months. The peak incidence occurs between 12 and 18 months. These seizures are typically triggered by a rapid increase in body temperature, often reaching 38°C or higher, in the absence of central nervous system infections or metabolic disturbances. While generally benign and self-limiting, they often cause significant concern among parents and caregivers due to their sudden onset and potential for recurrence. This recurrence may increase the risk of epilepsy, especially in complex cases (1−3).

Epidemiological studies have shown considerable regional variation in the incidence of febrile seizures. Globally, they affect approximately 2% to 5% of children (4−6) with similar rates observed in Western countries (2, 6). In some regions, such as Japan and Guam, the prevalence is significantly higher, ranging from 9% to 14% (1, 7), while in other regions, such as Hong Kong, the incidence is significantly lower (0.35%) (1).

Comprehensive national prevalence data on febrile seizures remain limited in Indonesia. However, local studies indicate that febrile seizures are a significant paediatric health concern. For instance, Kandou Hospital in Manado, Indonesia, reported 150 cases over two and a half years (8), and another study identified 417 cases in Pekanbaru over a similar period (9). In addition, Nurhayati et al. (10) documented 60 cases across three regional hospitals in Lampung Province in 2016.

Several risk factors contribute to febrile seizures and can be broadly categorised as environmental or genetic. Significant factors include age, family history of febrile seizures, elevated body temperature during fever, and the duration of a fever episode (11, 12). Children with a family history of febrile seizures are more likely to experience them, suggesting a genetic predisposition (12). Body temperature, particularly at the peak of a fever, is thought to play a major role in triggering febrile seizures (13). Other potential risk factors, such as anaemia and low birth weight, have also been proposed, although their roles are still being debated (14).

Despite these known associations, detailed research specific to Indonesia, particularly in Lampung Province, is limited. This study aims to address this knowledge gap by examining factors associated with febrile seizures in children under five years old at Abdul Moeloek Regional Hospital, the province’s central referral hospital. By identifying the key factors that contribute to these seizures, this research seeks to improve understanding of them and contribute to better management strategies for children at risk.

## Methods

### Study Design

This case−control study was conducted at Abdul Moeloek Regional Hospital in Lampung Province in December 2020. The case−control approach was chosen for its effectiveness in retrospectively identifying associations between febrile seizures and multiple potential risk factors. Children aged 6 to 60 months who presented with a fever during the study period were screened and classified into two groups: those who experienced a febrile seizure (cases) and those with a fever but no seizure (controls). The inclusion criteria were children aged 6 months to 60 months who were admitted with a fever and had complete medical records. Children with a history of seizures before 6 months of age, intracranial infections, long-term anticoagulant therapy, metabolic disorders, congenital diseases, or previous neurological disorders were excluded.

A total of 72 children who met the inclusion criteria were selected using consecutive sampling, with 36 children in each group. This technique ensured that all eligible participants during the study period were included, providing a representative sample of the target population. The minimum sample size was calculated using Epi Info™ software to detect an odds ratio (OR) of 4.0, with an alpha of 0.05, 80% power, and a 30% exposure proportion in the control group. This yielded a minimum requirement of 36 children per group (*n* = 72). Ethical approval for this study was obtained from the Health Ethics Committee of the Faculty of Medicine at Universitas Lampung (approval number: 3099/UN26.18/PP.05.02.00/2020). Written informed consent was obtained from all participating parents or caregivers.

### Data Collection

Data were collected through structured interviews and medical record reviews. Interviews with parents or caregivers were conducted to gather demographic and clinical data, including family history of febrile seizures, gender, birth weight, fever duration, fever intensity prior to hospitalisation, and previous seizure episodes.

Secondary data collected from the hospital’s medical records included information on body temperature during seizures, clinical diagnoses, and relevant clinical observations by attending physicians. Body temperature was assessed upon hospital admission by trained nursing staff using a digital axillary thermometer. The recorded temperature was obtained from the medical records and categorised as either 38°C to 39°C or > 39°C. Fever duration was classified as either ≤ 24 hours or > 24 hours. Age was grouped as either < 2 years or ≥ 2 years. Family seizure history and birth weight (< 2,500 grams or ≥ 2,500 grams) were also recorded. Anaemia status was determined based on haemoglobin levels documented in the medical records upon admission. Haemoglobin levels were measured using an automatic haematology analyser. Anaemia was defined as a haemoglobin concentration of < 11 g/dL, in accordance with World Health Organization guidelines for children under five years of age.

### Statistical Analysis

The Chi-square test was used to assess the association between febrile seizures and potential risk factors, with a significance threshold of *P* < 0.05. The OR was also calculated to measure the strength of the association between each risk factor and the occurrence of febrile seizures. Multivariate logistic regression analysis was used to evaluate the independent effects of significant factors. All statistical procedures were performed using SPSS version 23.0.

## Results

### Characteristics of Study Participants

The study included 72 children with fever, evenly divided between those who experienced febrile seizures (*n* = 36) and those who did not (*n* = 36). Most participants had body temperatures of 38°C to 39°C (77.8%), experienced fever durations exceeding 24 hours (61.1%), and were male (70.8%). Approximately 65.3% had a family history of seizures, 43.1% had low birth weight (< 2,500 g), and 55.6% were anaemic (Table 1).

### Factors Associated with Febrile Seizures

Significant associations were identified between febrile seizures and the following factors: body temperature > 39°C (*P =* 0.047, OR = 4.00), fever duration ≤ 24 hours (*P =* 0.030, OR = 3.35), male gender (*P =* 0.010, OR = 4.96), positive family history of seizures (*P =* 0.001, OR = 7.75), and birth weight < 2,500 grams (*P =* 0.017, OR = 3.64). Neither anaemia (*P =* 0.097, OR = 2.50) nor age (*P =* 0.604, OR = 1.50) demonstrated significant associations (Table 2).

Multivariate logistic regression analysis identified the following as independently associated with the occurrence of febrile seizures: family history (adjusted OR = 9.83; 95% CI: 2.68, 36.12; *P =* 0.001), male gender (adjusted OR = 7.06; 95% CI: 1.82, 27.36; *P =* 0.005), and body temperature > 39°C (adjusted OR = 4.60; 95% CI: 1.04, 20.41; *P =* 0.045). In contrast, birth weight and fever duration showed no independent significance. The model explained 43.3% of the variance in the incidence of febrile seizures (Nagelkerke *R*^2^ = 0.433), demonstrated good calibration (Hosmer–Lemeshow χ^2^ = 3.31, *P =* 0.914) and showed strong discriminative ability (AUC = 0.853), with an overall classification accuracy of 75% (Table 3).

## Discussion

This study reinforces the multifactorial nature of febrile seizures and contributes to our understanding of risk stratification in clinical settings. Our findings demonstrate that several factors, including body temperature during fever, fever duration, male gender, family history of seizures, and low birth weight, are significantly associated with an increased risk of febrile seizures. While these results are consistent with previous studies, some findings diverge, suggesting that regional or population-specific factors may influence the occurrence of febrile seizures.

In this study, children with fevers exceeding 39°C were more likely to experience febrile seizures. This finding is consistent with other studies that have identified a significant association between high fever and seizure onset (15, 16). Elevated body temperature may increase the brain’s metabolic demands, potentially leading to transient hypoxia and disrupted neuronal activity, mechanisms that may lower the seizure threshold (17). In our study, the risk of febrile seizures increased approximately four times in children with fevers exceeding 39°C, which is consistent with prior findings (18). These findings indicate the importance of managing fevers in young children, especially when temperatures exceed 39°C.

Similarly, we found a significant association between shorter fever duration (< 24 hours) and the occurrence of febrile seizures. These results align with previous literature indicating that febrile seizures frequently occur during the early phase of fever (19, 20). A rapid escalation of fever could be a critical trigger for febrile seizures, emphasising the importance of early intervention.

Our findings also indicate that male children are more likely to experience febrile seizures than females. This is consistent with many other studies (8, 21). Although the exact reasons for this gender difference are not fully understood, it may be related to biological factors, such as differences in immune responses, hormonal influences, or genetic predispositions (22). Males may be more susceptible to infections or have different immune responses, which may make them more prone to febrile seizures when they have a fever. Furthermore, recent insights into the sex-specific expression of immune genes and their interaction with fever-induced cytokine storms suggest that males may exhibit heightened neuroinflammatory responses during febrile illness, thereby increasing their susceptibility to seizures (23).

A positive family history of seizures was one of the strongest predictors of febrile seizures in our study. Children with a positive family history were 7.75 times more likely to experience febrile seizures, which is consistent with previous studies (24, 25). Specific genetic loci have been associated with increased susceptibility, and our data further support the heritable component of febrile seizures (14). Our study further supports the role of genetic factors in the aetiology of febrile seizures, suggesting that clinicians should be aware of familial risks when managing febrile children.

Although birth weight was not an independent predictor in our multivariate model, its initial association may reflect the impact of perinatal complications, such as hypoxia-ischaemia, intraventricular haemorrhage, and subsequent neurodevelopmental immaturity. These conditions can sensitise neural networks and lower the seizure threshold in response to febrile episodes. Notably, neonatal MRI findings in low birth weight infants have shown white matter vulnerabilities and delayed myelination, conditions associated with increased seizure susceptibility (26).

Although anaemia was not statistically significant in our study, it is still biologically plausible that it contributes to the condition. Iron plays vital roles in neurotransmitter synthesis (particularly of γ-aminobutyric acid [GABA] and dopamine), mitochondrial energy metabolism, and myelin production. Iron deficiency may impair these processes, which could influence seizure thresholds during systemic stressors, such as fever. The inconclusive associations across studies warrant further investigation with standardised anaemia definitions and longitudinal iron status assessments (27).

Our multivariate model explained 43.3% of the variance in febrile seizure risk, indicating the presence of additional unmeasured or interacting variables. Future studies should explore the role of other emerging risk factors, such as epigenetic modifications, gut−brain axis interactions (particularly microbial metabolites), and inflammatory cytokine profiles, in children with and without febrile seizures. Investigating postictal neuroinflammatory biomarkers can also provide insight into long-term neurodevelopmental outcomes.

Our study has several limitations. The case–control design limits our ability to draw causal inferences, and selection bias may have occurred due to the study’s setting at a single hospital. In addition, relying on parental recall for certain variables (e.g., fever duration and family history) may have led to reporting bias. Future longitudinal studies are needed to validate these findings and to elucidate further the biological mechanisms underlying febrile seizures. Moreover, genomic and molecular investigations may increase our understanding of the pathophysiology and facilitate targeted interventions.

## Conclusion

Febrile seizures are significantly associated with male sex, elevated body temperature, and family history. Awareness of these risk factors can lead to earlier recognition and management, which can ultimately improve paediatric neurological health.

## Figures and Tables

**Table 1:** Characteristics of study participants (*n* = 72)

Variable	*n*	%
**Febrile seizure**		
Yes	36	50
No	36	50
**Body temperature during fever**		
38°C to 39°C	56	77.8
> 39°C	16	22.2
**Duration of fever**		
≤ 24 hours	28	38.9
> 24 hours	44	61.1
**Gender**		
Male	51	70.8
Female	21	29.2
**Age**		
< 2 years	21	29.2
> 2 years	51	70.8
**Family history of seizure**		
Yes	47	65.3
No	25	34.7
**Birth weight**		
< 2,500 grams	31	43.1
> 2,500 grams	41	56.9
**Anaemia**		
Yes	40	55.6
No	32	44.4

**Table 2:** Crude odds ratio for factors associated with febrile seizures among children

Variable	Febrile seizure		*P*	OR (95% CI)

	Yes *n* (%)	No *n* (%)		
**Body temperature**				
> 39° C	12 (33.3)	4 (11.1)	0.047*	4.00 (1.15, 13.95)
38°C to 39°C	24 (66.7)	32 (88.9)		
**Duration of fever**				
≤ 24 hours	19 (52.8)	9 (25.0)	0.030*	3.35 (1.24, 9.10)
> 24 hours	17 (47.2)	27 (75.0)		
**Gender**				
Male	31 (86.1)	20 (55.6)	0.010*	4.96 (1.57, 15.68)
Female	5 (13.9)	16 (44.4)		
**Age**				
< 2 years	12 (33.3)	9 (25.0)	0.604	1.50 (0.54, 4.19)
≥ 2 years	24 (66.7)	27 (75.0)		
**Family history of seizure**				
Yes	31 (86.1)	16 (44.4)	0.001*	7.75 (2.45, 24.49)
No	5 (13.9)	20 (55.6)		
**Birth weight**				
< 2,500 grams	21 (58.3)	10 (27.8)	0.017*	3.64 (1.36, 9.75)
≥ 2,500 grams	36 (41.7)	36 (72.2)		
**Anaemia**				
Yes	24 (66.7)	16 (44.4)	0.097	2.50 (0.96, 6.49)
No	12 (33.3)	20 (55.6)		

* Significant associations were indicated by Chi-square test at *P* < 0.05

**Table 3:** Multiple logistic regression for factors associated with febrile seizures in children

Variable	Adjusted OR (95% CI)	*P-*value
Family history of seizure:		
Yes, No (reference)	9.83 (2.68, 36.12)	0.001
Gender:		
Male, Female (reference)	7.06 (1.82, 27.36)	0.005
Body temperature:		
> 39°C, 38°C to 39°C (reference)	4.60 (1.04, 20.41)	0.045

Model fit:Omnibus test, *P* = 0.033; Hosmer–Lemeshow χ^2^ = 3.31, *P* = 0.914; Nagelkerke *R^2^* = 0.433; AUC = 0.853; Classification accuracy = 75%
